# *Allium aralii* (Amaryllidaceae, sect. *Codonoprasum*), a New Species from Southeastern Anatolia (Türkiye) Based on Morphological Characters and Phylogenetic Evidence

**DOI:** 10.3390/plants15101574

**Published:** 2026-05-21

**Authors:** Mehmet Maruf Balos, Yavuz Bülent Köse, Veysel Sonay, Fatmanur Tunç

**Affiliations:** 1 Department of Pharmaceutical Botany, Faculty of Pharmacy, Harran University, Haliliye, 63050 Şanlıurfa, Türkiye; mbalos@gmail.com; 2 Department of Pharmaceutical Botany, Faculty of Pharmacy, Anadolu University, 26470 Eskişehir, Türkiye; fatmanurt@anadolu.edu.tr; 3 Graduate Education Institute, Harran University, Haliliye, 63050 Şanlıurfa, Türkiye; sonayveysel@gmail.com

**Keywords:** *Allium*, endemic, sect. *Codonoprasum*, morphology, taxonomy, phylogeny, seed micromorphology

## Abstract

*Allium aralii* Balos, Köse & Sonay *sp. nov.* (Amaryllidaceae, sect. *Codonoprasum*) is described as a new species from southeastern Anatolia, Türkiye. The species is morphologically distinguished from its closest relatives—*A. euphraticum*, *A. turcicum* subsp. *turcicum*, *A. turcicum* subsp. *fusciflorum*, and *A. yilandaghense*—by a unique combination of characters: blackish outer bulb tunics, semi-cylindrical solid leaves exceeding the inflorescence, an extremely long persistent two-valved spathe (4.5–28.0 cm), a lax hemispherical inflorescence (3–4 cm diam., 10–70 flowers), a goblet-shaped perigon with dentate inner tepals, exserted bicolored stamens (white at base and apex, purple in the middle), a reticulate-foveate ovary, and verrucate seed ornamentation with undulate anticlinal walls. Seed micromorphology examined by scanning electron microscopy (SEM) further supports species delimitation. Molecular phylogenetic analyses based on nuclear ITS and chloroplast matK sequences place *A. aralii* within a well-supported clade containing *A. turcicum* and allied species, corresponding to the eastern Mediterranean lineage of sect. *Codonoprasum*. ITS genetic distances between *A. aralii* and its morphologically closest relatives range 0.0632, falling within the typical interspecific range for *Allium*. The species is known from a single locality in Bozova district (Şanlıurfa) with fewer than 100 mature individuals and is assessed as Critically Endangered (CR) according to IUCN criteria. This discovery highlights southeastern Anatolia as an underexplored center of *Allium* diversification and underscores the importance of integrative taxonomy for species delimitation within this taxonomically complex group.

## 1. Introduction

The genus *Allium* L. represents one of the most species-rich groups of petaloid monocots and is the type genus of the subfamily Allioideae (Amaryllidaceae) [1]. According to the current phylogenetic framework supported by molecular data, the genus is subdivided into 15 subgenera and over 80 sections, which are organized into three major evolutionary lineages [2,3,4]. Currently, *Allium* comprises approximately 1089 recognized species [5]. Its distribution is predominantly Holarctic, with the majority of taxa occurring in the Northern Hemisphere, particularly in arid and semi-arid regions characterized by seasonal drought. The primary center of diversity is situated in Southwest and Central Asia, while North America represents a secondary center of speciation [6].

*Allium* sect. *Codonoprasum* Rchb. is one of the largest and taxonomically most complex groups within the genus *Allium*, consisting of perennial bulbous plants that are mainly distributed across the Old World, with centers of diversity in Southwest Asia, as well as in the Mediterranean Basin [7]. Members of this section are usually characterized by narrow, flat, semi-cylindrical, or channeled leaves; a conspicuous involucre (spathe) subtending an umbellate inflorescence; and campanulate to subglobose flowers [8,9]. Species of sect. *Codonoprasum* typically occur in arid, steppic, rocky, or mountainous environments; notably, a high rate of speciation has been observed in Anatolia, which was previously recognized as a biodiversity hotspot.

According to the Illustrated Flora of Türkiye, the latest comprehensive revision of *Allium* by Koyuncu et al. recognized 225 species in Türkiye [10]. Subsequent studies have identified additional species, increasing this number to 240 [11,12]. With the discovery of *A. aralii*, the number of *Allium* species in Türkiye reaches 241.

The eastern and southeastern Anatolia regions are less studied from a floristic perspective, and especially in terms of the *Allium* genus. As a result of floristic studies conducted in these regions between 2021 and 2026 (January), 17 new *Allium* species have been identified and one new record [13,14,15,16,17,18,19,20,21,22,23,24,25,26,27,28,29,30].

In June 2024, during field trips in Bozova district (Şanlıurfa/Southeast Türkiye) by the authors, and together with nature photographer and local nature researcher Şükrü Balıkçı, some unusual *Allium* specimens were observed. Approximately 20 specimens were collected from one location within the plant’s natural range. Detailed morphological analyses demonstrated that these specimens represent an undescribed species.

Over the past two decades, phylogenetic studies have significantly reshaped the infrageneric classification of *Allium*. Early molecular phylogenetic research—primarily utilizing nuclear ribosomal internal transcribed spacer (ITS) and chloroplast (cp) regions—identified three major lineages comprising 15 subgenera and 72 sections [3,6]. Subsequent studies have provided crucial insights into the evolutionary processes and taxonomy of individual subgenera, such as subgenera *Melanocrommyum* Webb et Berthel. [31,32], *Anguinum* (G. Don. ex W.D.J. Koch) N. Friesen [33,34], and *Amerallium* (Traub) Kamelin [35], as well as specific sections including *Cepa* (Mill.) Prokh. [36], *Allium* L. [37], and *Rhizirideum* (G.Don ex Koch) Wendelbo [38]. The most comprehensive phylogeny of *Allium* to date, published by Han et al. [39], investigated 448 species using eight plastid loci and one nuclear locus to examine the role of polyploidy in species diversification. Despite these advances, significant knowledge gaps persist, particularly regarding species-rich and taxonomically complex subgenera where traditional morphology-based classifications have failed to provide an adequate organizational framework [40,41].

Section *Codonoprasum* Reichenb., which belongs to the third evolutionary lineage, is one of the most diverse sections within the subgenus *Allium*, the largest subgenus of the genus *Allium.* The center of its diversity extends from the Mediterranean region and into western Iran—Turan [9,42,43]. This section now has about 160 valid species; many of which are polyploid. Due to the fact that *Codonoprasum* is relatively morphologically homogenous, it suffers from a complex and frequently inconsistent classification system, and poorly understood evolutionary relationships between its members. These two issues have resulted in a large number of past, and ongoing, naming issues for species described as new from different regions [5,7]. To aid classification, sect. *Codonoprasum* has been divided into several subsections—such as subsections *Codonoprasum*, *Haemoprasum* (F. Herm.) Wendelbo, *Longistamineum* (F. Herm.) Wendelbo, and *Phalerea* (F. Herm.) Wendelbo—as well as informal groups (e.g., the *A. paniculatum*, *A. carinatum*, and *A. stamineum* assemblages); however, these morphological subdivisions have not yet provided adequate information for establishing evolutionary relationships [44,45,46].

Molecular phylogenetic analyses dealing specifically with section *Codonoprasum* have to date only been done on an extremely small number of species. An unpublished thesis that was briefly summarized by Salmeri et al. [46] looked at 31 species using molecular data from ITS and two plastid loci (psbA-trnH, rbcL) and provided confirmation of monophyly for this section but left the relationship between deep nodes unresolved. Subsequently, a study by Salmeri et al. [46] analyzed 18 species and identified two monophyletic clades in this section. The most thorough phylogenetic analysis conducted to date was recently published [41] and examined 48 taxa (approximately 30% of the total diversity of the section) across three genomic loci (nrITS, trnH-psbA, and trnL-ndhJ) with multiple accessions for each taxon. The findings provide strong evidence of the monophyly of the sect. *Codonoprasum* closely related to sect. *Cupanioscordum* Cheshm. Additionally, Duchoslav et al. [41] identified five major lineages or clades (I–V) based on their data, with early diverging clades I and II being restricted to the eastern Mediterranean (Levant), Türkiye, the Caucasus, and Crimea, indicating an eastern origin to this section followed by westward dispersal of its members. Furthermore, their phylogenetic results show that the traditionally recognized subgroups (subsections) are not monophyletic, and that morphologically defined species complexes (e.g., *A. paniculatum* clade, *A. carinatum* clade; *A. flavum* clade) exhibit complex patterns of incomplete lineage sorting, polyploidy and hybridization [41]. The mapping of genome size (1Cx values) to the phylogenetic trees also demonstrates a punctuated mode of evolution and a significant downsize in genome sizes of polyploids among most of clade members.

Duchoslav et al. [41] examined a larger number of taxa than previously; however, they did not study many Turkish regions, and grossly neglected eastern and southeastern Anatolia, which is a region that has not been floristically documented well. This is a gap in the study as Anatolia as a whole is a globally recognized biodiversity hotspot with strong representation for *Allium* taxa. This serves to illustrate the vastness of the still poorly understood *Allium* diversity in this area [13,14,15,16,17,18,19,20,21,22,23,24,25,26,27,28,29,30]. Phylogenetic study to date has afforded particular consideration to sect. *Codonoprasum* taxa from Türkiye; only a single species from Türkiye in this section has been accessed with supporting molecular phylogenetic evidence [41].

This study describes a new species of *Allium* from Türkiye based on a combination of morphological characteristics (morphology) and seed morphology, and selected DNA barcoding markers. In doing so, this study lays a vital groundwork for further systematic and phylogenetically-based taxonomy of *Allium* sect. *Codonoprasum* in both Türkiye and the broader eastern Mediterranean region.

## 2. Results

### 2.1. Taxonomy

**Figure 1:**
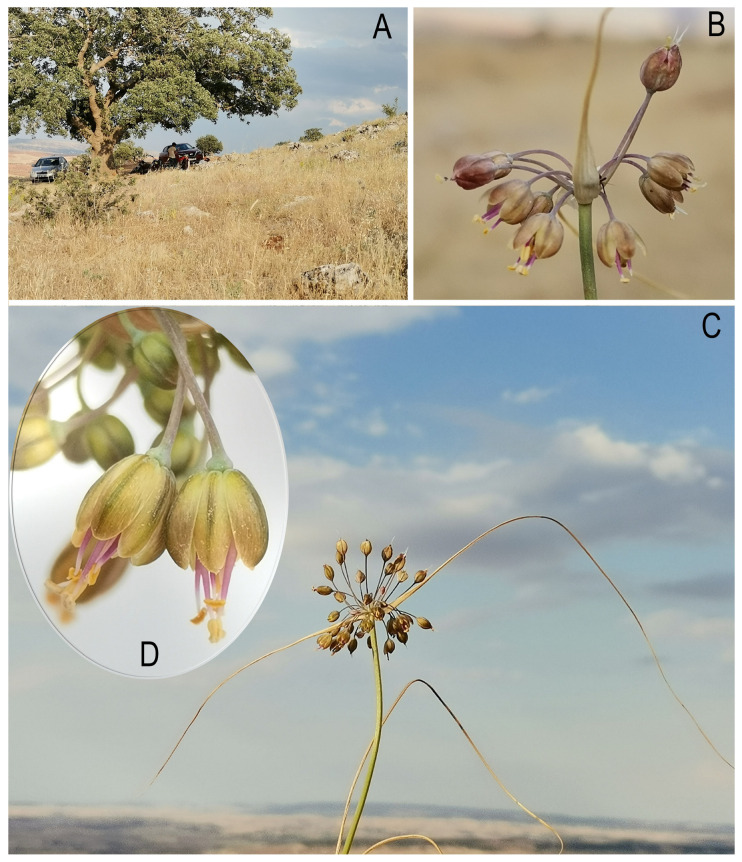
Habit and reproductive morphology of *Allium aralii*. (**A**) With oak trees on Mount Kaplandağı. (**B**,**C**) Inflorescences showing lax hemispherical structure. (**D**) Detail of perigon and pedicel, illustrating tepal arrangement and coloration.

**Figure 2:**
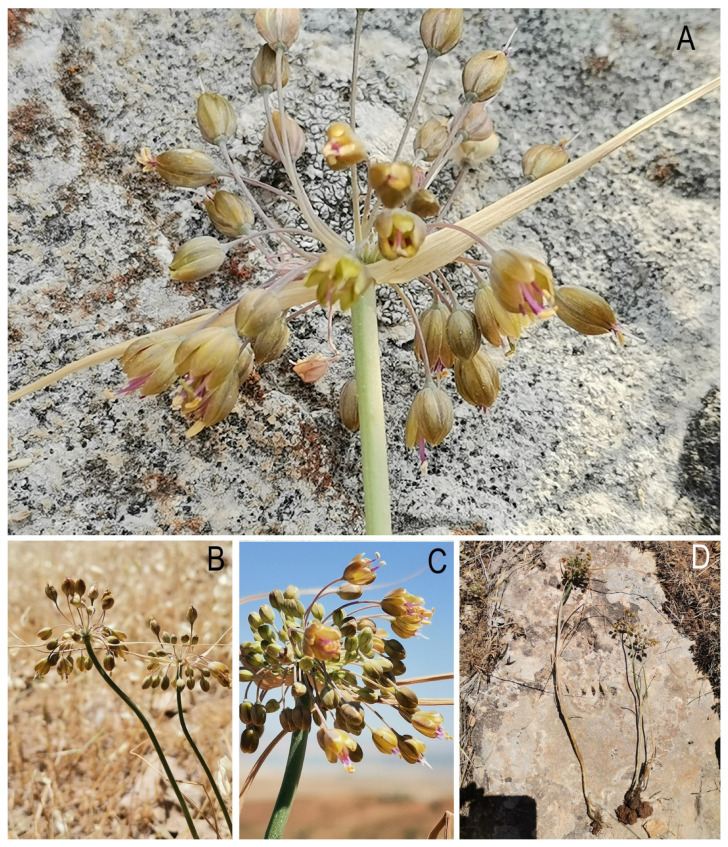
Additional views of *Allium aralii*. (**A**–**C**) Variation in inflorescence morphology, highlighting density and flower arrangement. (**D**) Whole plant habit, showing relative proportions of leaves, scape, and inflorescence.

**Figure 3:**
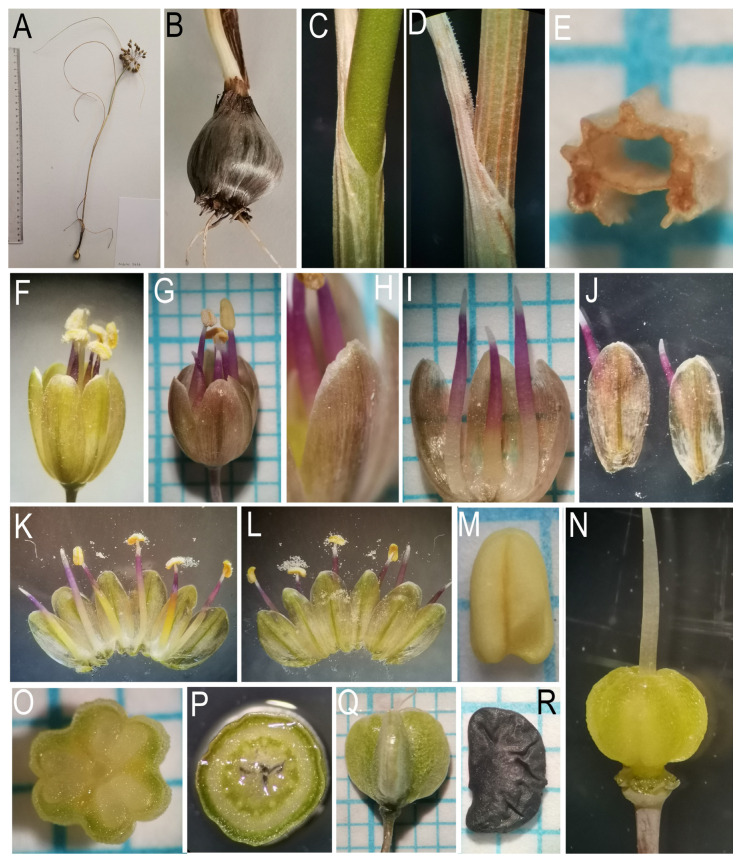
*Allium aralii* (From holotype M. Balos 5616). (**A**) Habit. (**B**) Bulb. (**C**,**D**) Leaf sheathing and stem. (**E**) Leaf cross-section. (**F**,**G**) Perigon. (**H**) Close-up view of the outer tepal. (**I**) Inner surface of open perigon and filament. (**J**) Outer–inner–outer tepal. (**K**) Inner surface of open perigon. (**L**) Outer surface of open perigon. (**M**) Anther (**N**) Ovary. (**O**) Ovary cross-section (**P**) Scape cross-section. (**Q**) Capsule. (**R**) Seed.

*Allium aralii* Balos, Köse & Sonay *sp. nov.* (Figure 1, Figure 2 and Figure 3).

**Figure 4:**
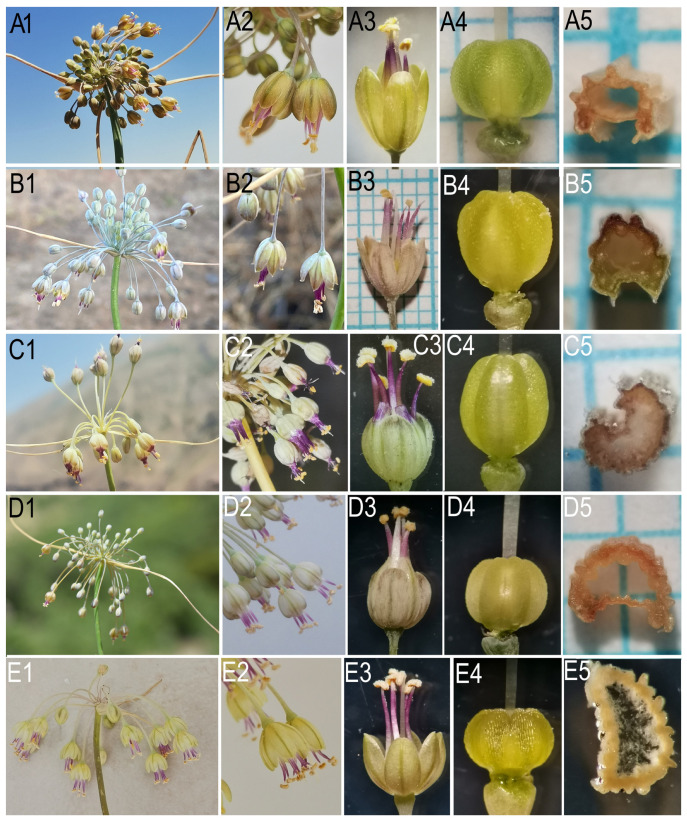
Comparative morphology of *Allium aralii* and related taxa. (**A1**–**A5**) *A. aralii* (From holotype, M. Balos 5616); (**B1**–**B5**) *A. euphraticum*; (**C1**–**C5**) *A. turcicum* subsp. *turcicum*; (**D1**–**D5**) *A. turcicum* subsp. *fusciflorum*; (**E1**–**E5**) *A. yilandaghense*. (1) Inflorescence; (2) perigon and pedicel; (3) perigon; (4) ovary; (5) leaf cross-section. Note diagnostic differences in perigon shape, ovary structure, and leaf anatomy.

Diagnosis: *Allium aralii* belongs to *Allium* sect. *Codonoprasum* and is morphologically distinguished from its closely related species—*A. euphraticum*, *A. turcicum* subsp. *turcicum*, and *A. turcicum* subsp. *fusciflorum*—by the following combination of characters: outer bulb tunics blackish (vs. brownish in *A. euphraticum* and *A. turcicum* subsp. *turcicum*, brownish-black in *A. turcicum* subsp. *fusciflorum*); bulbs 1.0–1.5 × 0.8–1.0 cm (vs. 1–2 cm in *A. euphraticum*, 1–2 cm in *A. turcicum* subsp. *turcicum*, 1–2 cm in *A. turcicum* subsp. *fusciflorum*); scape slender, 16–30 cm long (vs. 14–44 cm in *A. euphraticum*, 10–40 cm in *A. turcicum* subsp. *turcicum* and subsp. *fusciflorum*); leaves semi-cylindrical, solid, exceeding the inflorescence, 6.5–21.0 × 0.08–0.1 cm (vs. filiform, hollow, exceeding the inflorescence in *A. euphraticum*, or semi-cylindrical but not exceeding in *A. turcicum* subsp. *turcicum*); spathe valves very long, 4.5–28.0 cm, strongly exceeding the inflorescence (vs. long but proportionally shorter); inflorescence lax, hemispherical, 3–4 cm in diam., with 10–70 flowers (vs. lax in all, but hemispherical vs. campanulate or globose); perigon goblet-shaped (vs. campanulate in *A. euphraticum* and *A. turcicum* subsp. *turcicum*, globose in *A. turcicum* subsp. *fusciflorum*); tepals 4.0–5.0 mm long, dark yellowish-brown to greenish-brown, inner tepals with dentate apex (vs. mucronate in *A. euphraticum*, irregularly dentate in *A. turcicum* subsp. *turcicum*); stamens distinctly exserted, filaments 3.5–4.0 mm long, white at base and apex, purple in the middle (vs. upper part purplish in the compared species, with filaments 5.5–6.0 mm in *A. euphraticum*); ovary reticulate-foveate, 1.5–2.0 × 2.0–2.4 mm (vs. reticulate-papillose in *A. euphraticum*, reticulate in *A. turcicum* subsp. *turcicum*, papillose in *A. turcicum* subsp. *fusciflorum*) (Figure 4).

*Type*: Türkiye. Şanlıurfa: Bozova, Mt. Kaplandağı, oak and rocky area, 37°20′0.25″ N, 38°34′11.06″ E, 840 m, 25 May 2024, M. Balos 5616 (holotype: HUEH; isotypes: ESSE). Ibid, 24 May 2025, M. Balos 5682 (Paratype HUEH).

Description: Perennial, bulbous plant. Bulb ovoid, 1.0–1.5 × 0.8–1.0 cm; a single scape emerges from bulb; outer tunics papyraceous, blackish; inner tunics membranous, off-white; without bulblets; scape 16–30 × 0.1–0.3 cm, cylindrical, solid, erect or flexible, glabrous; usually covered with leaf sheaths that make up 2/3–3/4 of its total length. Leaves 2–4, 6.5–21.0 cm × 0.8–1.0 mm, semi-cylindrical, canaliculate, solid, exceed inflorescence. Leaves margin and sheaths scabrid. Spathe persistent with two unequal valves, very exceeds to inflorescence; narrowly lanceolate at base and filiform towards apex; base width ca. 4–7 mm; 4–6 nerved, entire margins. Short valve 3–16 cm, the longer valve 4.5–28.0 cm. Inflorescence hemispherical, lax, 3.0–4.0 cm in diam., with 10–70 flowers, pedicels unequal, 1.0–2.5 cm length, bracteolate, brown. Perigon goblet-shaped, tepals oblong, naviculate, dark yellowish-brown or greenish-brown, dark brownish or greenish midveins. Tepals ±equal, outer 4.0–5.0 × 2.0–2.5 mm, inner 4.0–5 × 1.8–2.0 mm, outer tepal elliptic-ovate, subacute to rounded, inner tepal elliptic-ovate, subacute to rounded, dentate apex. Stamens distinctly exserted; filaments simple, white at base and apex, purple in the middle, subulate at apex, almost equal, simple, some inner perigon filament lower part is yellow; 3.5–4.0 mm long, connate at the base into an annulus that is 0.9–1.0 mm high; anthers yellow, 1.2–1.8 × 0.6–0.8 mm, oblong, rounded apex. Ovary green, globose or ellipsoid, reticulate-foveate, with furrow channeled, 1.5–2.0 × 2.0–2.4 mm, with 0.3–0.5 mm short stipe; style equal or longer than anthers, 3.5–5.5 mm length, white, stigma tip blunt. Capsule trivalved, globose; 4.0 × 4.5 mm, valves obcordate, emarginate, sessile, 4.5 × 4.0 mm, seed hemispheroidal, surface reticulate, black, 3–4 × 2.1–2.1 mm (Table 1 and Table 2).

Phenology: Flowering and fruiting in May–June.

Etymology: The species name ‘aralii’ was given in honor of the extraordinary academic achievements of the second author’s son, Aral Köse. Aral is an exceptional student who, after pursuing a dual degree in Electrical and Electronics Engineering and Mathematics at Boğaziçi University, has been awarded a full scholarship for a PhD program at the University of California, Santa Barbara (UCSB).

Turkish common name of the new species: *Allium* is called “Soğan” in Turkish. The author proposes “Kaplandağı soğanı” as a vernacular name for *A. aralii* according to the guidelines of Menemen et al. [47].

Distribution and habitat: *Allium aralii* is endemic to Türkiye and distributed in the Irano-Turanian phytogeographical region. It grows on slopes and stony areas at elevations of 870 m and it is only known from the type locality (Bozova). Bozova district, where the newly discovered plant is located, is a region with a distinctive topographic structure and rich habitat diversity. Around 100 mature individuals, growing in two populations 200 m apart, were found between limestone cliffs, slopes and in deciduous shrubs. Associated species were *Achillea aleppica* DC., *Allium schergianum* Boiss., *Allium pseudoflavum* Vved., *Allium chrysantherum* Boiss. & Reut., *Ballota saxatilis* Sieber ex C.Presl, *Bromus squarrosus* L., *Bryonia alba* L., *Crataegus monogyna* Jacq., *Celtis tournefortii* Lam., *Prunus microcarpa* C.A.Mey., *Echinops spinosissimus* Turra, *Eryngium campestre* L., *Euphorbia cheiradenia* Boiss. & Hohen., *Haplophyllum buxbaumii* (Poir.) G.Don, *Hordeum bulbosum* L., *Teucrium polium* L., *Siebera pungens* (Lam.) J.Gay ex DC., *Lactuca praevia* C.D.Adams, *Lagoecia cuminoides* L., *Leiotulus secacul* (Mill.) Pimenov & Ostr., *Minuartia decipiens* (Fenzl) Bornm., *Nigella unguicularis* (Poir.) Spenn., *Papaver rhoeas* L., *Parietaria Judaica* L., *Picnomon acarna* (L.) Cass., *Podonosma orientalis* (L.) Feinbrun, *Quercus brantii* Lindl., *Torilis arvensis* (Huds.) Link, *Xeranthemum annuum* L., and *Verbascum glomeratum* Boiss.

Species conservation assessment: The new species was recorded in only one location with fewer than 100 adult individuals and an estimated habitat area (AOO) of less than 1 km^2^. It is likely also found in similar habitats in the immediate vicinity. However, although the distribution of the new species is in a mountainous area, natural risks threatening the plant have been observed in the region, such as ongoing sheep grazing pressure, quarrying activity, use of the area as a graveyard and picnic area, and drought due to climate change. Therefore, due to its limited population, number of locations (1), number of mature individuals, and the projected future decline in population and habitat quality, we propose that the species can be classified as Critically Endangered-CR [B1ab(i, ii, iii) + 2ab(i, ii, iii)] [48].

### 2.2. Seed Micromorphology

**Figure 5:**
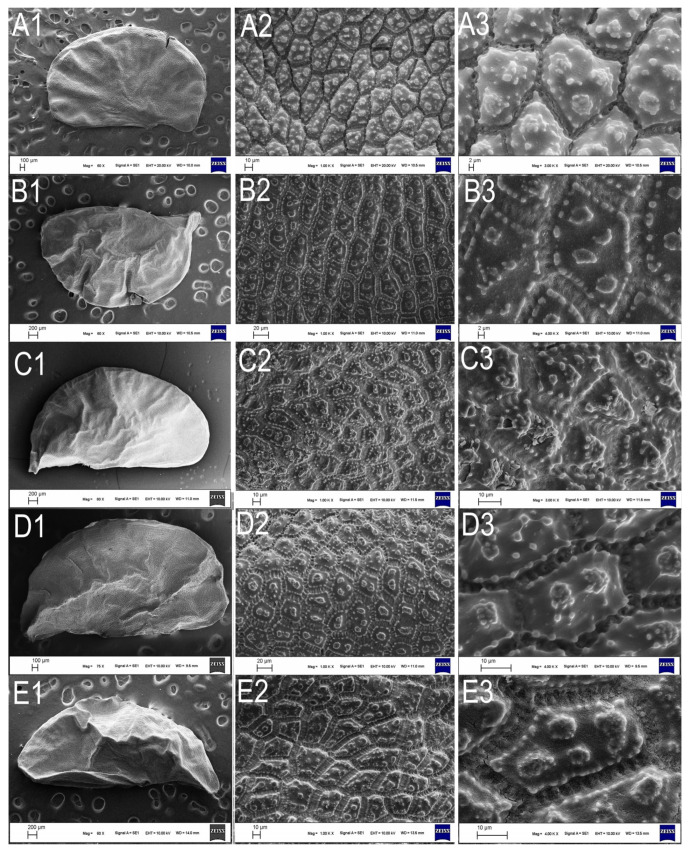
Seed micromorphology of *A. aralii* and related species under SEM. (**A1**–**A3**) *A. aralii*; (**B1**–**B3**) *A. euphraticum*; (**C1**–**C3**) *A. turcicum* subsp. *turcicum*; (**D1**–**D3**) *A. turcicum* subsp. *fusciflorum*; (**E1**–**E3**) *A. yilandaghense*. Images highlight differences in seed shape, testa cell shape, anticlinal wall pattern, and periclinal wall ornamentation, including the distinct verrucate surface characteristic of *A. aralii*. Magnification for column 1 (**A1**,**B1**,**C1**,**E1**) is 60× and (**D1**) is 75×, Magnification for column 2 (**A2**,**B2**,**C2**,**D2**,**E2**) is 1 Kx, Magnification for column 3 (**A3**,**C3**) is 3 Kx and (**B3**,**D3**,**E3**) is 4 Kx.

The micromorphological characters obtained from scanning electron microscopy of seed coats of *A. aralii* (Table 2; Figure 5(A1–A3)) demonstrate intraspecific consistency within *A. aralii* and distinct variability when compared to *A. euphraticum*, *A. turcicum* subsp. *fusciflorum*, and *A. yilandaghense*.

### 2.3. Phylogenetic Analysis

**Figure 6:**
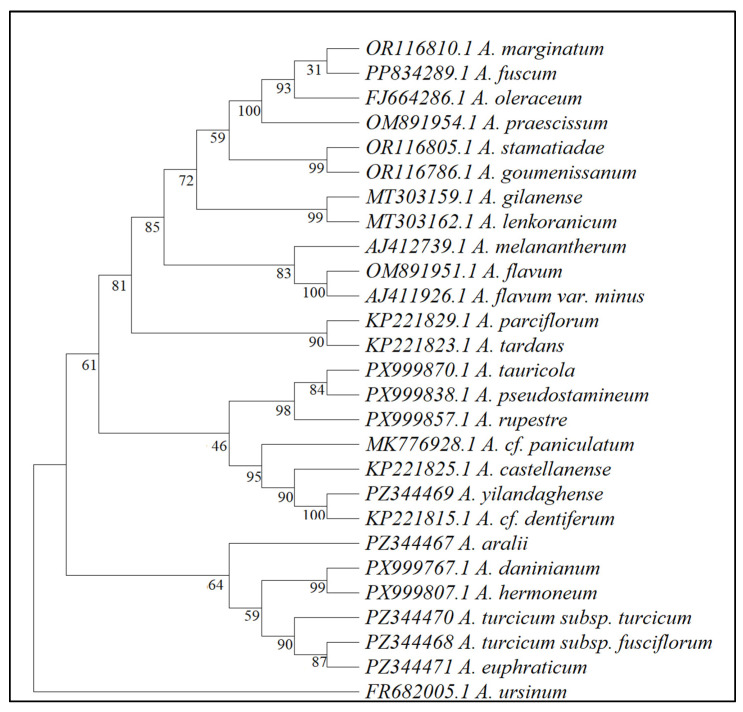
Phylogenetic tree (dendrogram) of selected *Allium* sect. *Codonoprasum* taxa based on ITS sequence data. Numbers at the nodes indicate bootstrap support values (%) derived from 1000 replications.

**Figure 7:**
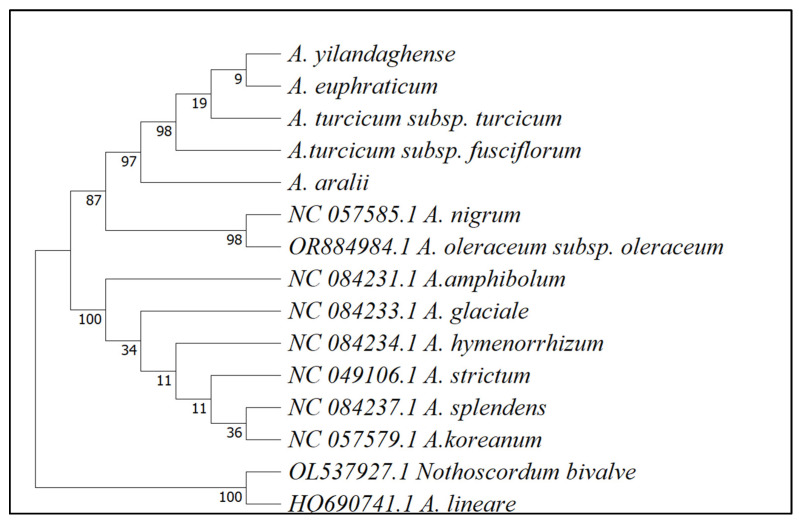
Phylogenetic tree (dendrogram) of selected *Allium* sect. *Codonoprasum* taxa based on matK sequence data. Numbers at the nodes indicate bootstrap support values (%) derived from 1000 replications.

The ITS dataset had 530 nucleotides and a total of 30 accessions represented in the final alignment (Figure 6) versus an alignment of 804 nucleotides for the matK dataset with 15 accessions (Figure 7). The Kimura 2-parameter model (K2P) was used to calculate pairwise genetic distance [49]. The genetic distance between *A. aralii* and its morphologically closest relatives was calculated based on the ITS region. The pairwise distance comparisons between *A. aralii* and other taxa can be seen in Table 3: to *A. turcicum* subsp. *turcicum* (0.063); to *A. euphraticum* (0.072); and to *A. turcicum* subsp. *fusciflorum* (0.076). In terms of the matK region, the calculated K2P distance between *A. aralii* and *A. turcicum* subsp. *fusciflorum* was 0.0088, or 0.88% (Table 4). The K2P distances from *A. aralii* to the other taxa were as follows: *A. euphraticum*, 0.0100; *A. yilandaghense*, 0.0100; and *A. turcicum* subsp. *turcicum*, 0.0113, which are equal to 1.00%, 1.00%, and 1.13%, respectively (Table 4). Among the compared taxa, the shortest distance (matK) was between *A. turcicum* subsp. *fusciflorum* and *A. yilandaghense* (0.012, 0.12%), and between *A. turcicum* subsp. *fusciflorum* and *A. euphraticum* (0.012, 0.12%). The longest matK distance was between *A. turcicum* subsp. *turcicum* and *A. oleraceum* subsp. *oleraceum* (0.0189 or 1.89%) (Table 4).

Maximum Likelihood (ML) analysis of 30 taxa using ITS data produced a well-resolved phylogeny (Figure 6). The ITS phylogeny places *A. aralii* (PZ344467) as basal to a clade that includes *A. daninianum*, *A. hermoneum,* and the *A. turcicum* complex (branch support = 64%). Within this neighboring group, *A. daninianum* and *A. hermoneum* form a sister subclade (branch support = 99%). This subclade is sister to a group containing *A. turcicum* subsp. *turcicum*, *A. turcicum* subsp. *fusciflorum* and *A. euphraticum* (branch support = 59%). *A. turcicum* subsp. *turcicum* is sister to a terminal group consisting of *A. turcicum* subsp. *fusciflorum* and *A. euphraticum* (branch support = 90%), and these two taxa share high branch support (branch support = 87%). This topology suggests that although *A. aralii* shares morphological affinities with the *A. turcicum* group, it occupies a distinct phylogenetic position as a basal lineage to the clades containing *A. daninianum/hermoneum* and *A. turcicum/euphraticum*. This indicates an early divergence within this eastern Mediterranean branch of sect. *Codonoprasum*.

The tree obtained using Maximum Likelihood (ML) method and matK sequences (804 bp, 15 taxa) is illustrated in Figure 7. *A. aralii* and *A. turcicum* subsp. *fusciflorum* were grouped together in a clade that contains both species from separate clades (94% bootstrap support) to form a monophyletic group with *A. aralii* based on 99% bootstrap support. The relationships among *A. turcicum* subsp. *turcicum*, *A. euphraticum*, and *A. yilandaghense* were not resolved with the low bootstrap percentage (13–23%). Other well-supported clades (≥70%) were *A. nigrum* being placed in a clade with *A. oleraceum* subsp. *oleraceum* (98% support), a large clade consisting of *A. amphibolum*, *A. glaciale*, *A. hymenorrhizum*, *A. strictum*, *A. splendens*, and *A. koreanum* (100% support), and finally, the outgroup to this analysis consisting of *Nothoscordum* bivalve and *A. lineare* (100% support) (Figure 7).

## 3. Discussion

The evaluation of diverse morphological features—including bulb morphology, leaf shape, flower structure, and seed coat microstructure—provides robust evidence to support the classification of *Allium aralii* as a distinct species within the taxonomically complex sect. *Codonoprasum* [9,42]. Compared to its close morphological relatives, such as *A. euphraticum* [23], *A. yilandaghense* [25], and both subspecies of *A. turcicum* (subsp. *turcicum* and subsp. *fusciflorum* [22]), *A. aralii* possesses a unique set of diagnostic features that distinguish it from these taxa (Table 1; Figure 1, Figure 2, Figure 3 and Figure 4).

The bulb tunics of *A. aralii* are one of the most immediately observable diagnostic characters. *A. euphraticum* and *A. turcicum* subsp. *turcicum* both have brownish outer tunics while *A. yilandaghense* has pale brown tunics [22,23,24,25]. In contrast, *A. aralii* is the only species to have blackened outer bulb tunics (Table 1). This blackened bulb tunic character is consistently observed among all of the gathered specimens and is therefore a reliable field character. The size and shape of the bulbs of *A. aralii* (1.0–1.5 cm × 0.8–1.0 cm) are similar to that of *A. euphraticum* (1–2 cm) and the *A. turcicum* subspecies; however, the bulbs of *A. aralii* are substantially less bulky than those of *A. yilandaghense* (2.5–3 cm). In addition to this, *A. aralii* does not produce any of the small bulblets that *A. yilandaghense* produces (1–3 bulblets per bulb), which have also been found to be taxonomically informative within sect. *Codonoprasum* [9,41]. This combination of bulb characters (blackish tunics, smaller bulb size, and absence of bulblets) clearly distinguishes *A. aralii* from *A. yilandaghense*, which has pale brown tunics, larger bulbs (2.5–3 cm), and produces 1–3 bulblets per bulb, highlighting the taxonomic significance of vegetative propagation traits within sect. *Codonoprasum* [50].

The scape morphology (16–30 cm, slender, cylindrical, solid) of *A. aralii* is well within the range of variability seen in the closely related taxa. However, the leaf morphology of *A. aralii* is remarkably different than that of the related taxa. The leaves of *A. aralii* are semi-cylindrical, solid, and are longer than their corresponding inflorescence (6.5–21 cm × 0.8–1 mm), whereas, the leaves of both *A. euphraticum* are filiform, hollow, and longer than their inflorescence [23] and those of *A. yilandaghense* are flat or semi-cylindrical and not longer than their inflorescence [25] (Table 1; Figure 4(A5–E5)). An analysis of the leaf cross-section (Figure 4(A5)) confirms the solid semi-cylindrical leaf morphology of *A. aralii*; however, these leaves exhibit scabrid, sheathed margins, which have also been reported to be taxonomically significant characters in *Codonoprasum* [8,43].

The morphology of the spathe (involucre) is also another important diagnostic complex. Within *Codonoprasum*, spathe characters have historically been considered important taxonomically [5,9]. *A. aralii* has a persistent, two-parted (compound) spathe that is exceptionally long, with valves measuring 3–16 cm (short valve) and 4.5–28 cm (long valve)—far exceeding the inflorescence length. The spathe valves are lanceolate and filiform at the apical end and have a very narrowly lanceolate base with four to six nerves and entire margins. The extreme elongation of the spathe valves provides a distinction for *A. aralii* among the other species compared in this study. Although *A. euphraticum* and the two subspecies of *A. turcicum* also have extremely long spathes [22,23], neither have the proportional length or persistence that are found in *A. aralii*. Although *A. yilandaghense* has spathe valves equal to or longer than the inflorescence, it does not exhibit the extreme elongation characteristic of *A. aralii* [25].

*A. aralii* inflorescences have a lax hemispherical shape, 3.0–4.0 cm in diameter, containing 10–70 flowers (Figure 1, Figure 2 and Figure 3). *A. turcicum* subsp. *fusciflorum* has a globose inflorescence, whereas *A. euphraticum*, *A. turcicum* subsp. *turcicum*, and *A. yilandaghense* have a hemispherical to ovoid inflorescence (Table 1). The most significant floral characteristics of *A. aralii* reside in its perigon and tepal morphology. *A. aralii* is unique within this group in possessing a goblet-shaped perigon; in contrast, related taxa exhibit campanulate (e.g., *A. euphraticum* and *A. turcicum* subsp. *turcicum*) or globose (e.g., *A. turcicum* subsp. *fusciflorum*) perigons [22,23,25] (Table 1; Figure 4A–E). Furthermore, *A. aralii* features oblong, navicular tepals (4.0–5.0 mm long) that are arranged more upright, forming a cup-like structure, unlike the more spreading perigons of closely allied species. The taxonomic significance of perigon shape within section *Codonoprasum* has been recently emphasized by [8,42].

Due to the recent taxonomic treatments of the section [45,46], changes in the shape and density of the inflorescence will be emphasized as important diagnostic characters by these authors. The pedicels were also different (1.0–2.5 cm), branched, and brown reflecting the overall morphology of sect. *Codonoprasum* [9].

The color of the tepal is typically dark yellowish-brown to greenish-brown with the midveins being slightly darker colored. One important feature that distinguishes *A. aralii* from the other taxa included in this comparison is that the inner tepals of *A. aralii* have dentate apices whereas the inner tepals of *A. euphraticum* have mucronate apices, the inner tepals of *A. turcicum* subsp. *turcicum* have irregularly dentate apices, and the inner tepals of *A. yilandaghense* have obtuse apices [22,23,25] (Table 1). Apical morphology of the tepal, particularly among the dentate and entire conditions, has been called upon as a useful diagnostic character in numerous *Allium* taxonomic publications [2,51].

*A. aralii* stamens have a distinct two-tone filament, with white at either end and purple in the center, which are long and protruding (3.5–4.0 mm). Some of the inner perigon filaments have yellowish tint below, creating a three-color scheme that is not found in the other species being compared. Although *A. euphraticum*, *A. turcicum* (subspecies), and *A*. *yilandaghense* also have stamens with protruding purple tips, the gradual white-purple-white transition seen in *A. aralii* is not seen in these three species [22,23,25]. Although filament color and protrusion have been observed to exhibit systematic significance in *Allium* [9,52], the filament bases (0.9–1.0 mm) are connate into an annulus, consistent with sect. *Codonoprasum* [8].

Anthers are yellow and measure 1.2 to 1.8 mm long and 0.6 to 0.8 mm wide and they have an elongated shape with a rounded tip (Figure 3L and Figure 4(A4)). The ovary of *A. aralii* is reticulate-foveate (Figure 3L and Figure 4(A4)), which represents a unique and defining micromorphological feature. In contrast, *A. euphraticum* and *A. yilandaghense* have reticulate-papillose ovaries; *A. turcicum* subsp. *turcicum* has a reticulate ovary and *A. turcicum* subsp. *fusciflorum* has a papillose ovary [22,23,25] (Table 1; Figure 4(B4–E4)). The reticulate-foveate form of the ovary, being comprised of a network of ridges enclosed by deep pits or depressions, has been established as being taxonomically significant in many *Allium* species; thus, it provides compelling evidence to support the distinction of *A. aralii* from its relatives. The style length of *A. aralii* ranges from 3.5 mm to 5.5 mm and is equal to or longer than that of the anther, is white in color, and has a bluntly pointed stigma. The capsule of *A. aralii* is globose (4.0 mm wide × 4.5 mm long) having two obcordate, emarginate valves, which supports its placement in the sect. *Codonoprasum* [9].

Using scanning electron microscopy to analyze seed coats has established a significant advance in species delimitation at the genus level. Seeds of *A. aralii* are oval-elliptic and range in size from 2.8–3.2 mm × 1.4–1.6 mm. This size is consistent with the size of seeds from *A. euphraticum* (length: 3.1–3.5; width: 1.9–2.2; L/W ≈ 1.5–1.6) and *A. turcicum* subsp. *fusciflorum* (length: 2.9–3.3; width: 1.5–1.7; L/W ≈ 1.9) [22,23]; however, *A. aralii* seeds exhibit an extremely narrow L/W ratio (1.9–2.0) compared to the L/W ratios of the other taxa. Additionally, the determination of seed size and shape as taxonomically useful within the *Allium* sect. *Codonoprasum* has been documented by some authors [52,53] (Table 2; Figure 5(A1–A3).

The shape of testa cells in *A. aralii* is elongated-polygonal, with a longitudinal orientation. The cell dimensions in *A. aralii* (45.5–62.0 × 22.0–34.5 µm) are similar to those of other closely related species; however, the cells of *A. aralii* possess clearly defined undulations at the margins, with anticlinal wall thickness measuring 3.2–4.8 µm. This undulatory pattern, characterized by thickened wall regions, differs from the sinuate-undulatory pattern observed in *A. euphraticum* (3.5–5.2 µm). Moreover, it is less pronounced than the straight and curved patterns found in both *A. turcicum* subsp. *turcicum* (2.5–4.0 µm) and *A. yilandaghense* [22,23,25] (Table 2). The morphology and thickness of anticlinal walls are considered important diagnostic characters in seed coat studies of *Allium* species [52,54,55].

Among these five species, the most notable distinction is in their periclinal wall ornamentation. While *A. aralii* is characterized by convex periclinal walls adorned with verrucate shaped ornamentation (small warty protuberances), *A. euphraticum* has larger verrucate ornamentation. In contrast, *A. turcicum* subsp. *turcicum* is noted for having smooth striated periclinal walls and lacking any observable verrucate ornamentation. Additionally, although *A. turcicum* subsp. *fusciflorum* still contains verrucate ornamentation; it is present in a more angular pattern than that of *A. aralii*. Finally, while the wall structure of *A. yilandaghense* is also similar to that of *A. aralii*, it has a distinctly rugose and verrucate shaped profile [22,23,25] (Table 2; Figure 4). The periclinal wall ornamentation, either verrucate, rugose, striated, or granular type, has previously been established to be species-specific, in many *Allium* groups [51,54,55,56,57,58]. Only *A. aralii* exhibits both the combination of verrucate wall ornamentation along with undulating anti-clinal boundaries and convex periclinal walls relative to the other taxa examined in this study.

As molecular barcoding tools, the ribosomal internal transcribed spacer (ITS) and the chloroplast matK region have become popular choices for assessing evolutionary relationships and defining species boundaries within *Allium* [3,6]. ITS is often used as the first choice for infrageneric taxonomy due to its fast evolutionary rate and its ability to be resolved at the species level. In addition, matK is a significant DNA barcoding marker for terrestrial plants because of its capacity to discriminate species with precision and its simplicity in obtaining genetic sequences [57].

Recently, a broad phylogenetic study of the sect. *Codonoprasum* by Duchoslav et al. [41] defined a basis from which to interpret our results. Duchoslav et al. [41] reported on a phylogenetic study involving 48 taxa (approximately 30% of the section’s total diversity) that utilized three genes (nrITS, trnH-psbA, and trnL-ndhJ) and, through the analysis of these three gene sets, established five major lineages (Clades I through V) among the members of this section. Also, the results of the study suggest that the traditionally recognized subsections within the sect. *Codonoprasum* do not form monophyletic lineages in the molecular phylogeny [41]. Of particular importance is that Clades I and II, the early-diverged clades, are found to occur only in the eastern Mediterranean region, including the Levant, Türkiye, the Caucasus, and Crimea; this suggests a possible eastern location of origin and subsequent movement to the west.

The ITS analysis revealed that *A. aralii* (PZ344467) belongs to the eastern Mediterranean group containing sect. *Codonoprasum* as described by Duchoslav et al. (2026) [41], which falls under Clade I per their criteria. Based on the relationships shown by the trees used for this analysis, *A. aralii* is in a basal position within a well-supported group (BS = 64%) that includes other taxa from the regional area such as *A. daninianum*, *A. hermoneum*, and what we call the *A. turcicum* group. Within this well-supported association, *A. daninianum* (PX999767.1) and *A. hermoneum* (PX999807.1) comprise a sister pair to one another with maximum support (BS = 99%), and they are both directly related to *A. turcicum* subsp. *turcicum*, *A. turcicum* subsp. *fusciflorum*, and *A. euphraticum* (BS = 59%). The placement of *A. aralii* so far below the rest of these lineages suggests that this taxon represents a genetically distinct species that diverged relatively early during the evolution of the Clade I group. The pairwise ITS distances calculated between *A. aralii* and its relatives (0.063–0.078) are within the typical range of interspecific divergence documented for the genus *Allium*, thereby supporting the recognition of *A. aralii* as a distinct species within the Anatolian–eastern Mediterranean branch of sect. *Codonoprasum*.

Additionally, we observed an unexpected relationship between *A. yilandaghense* (PZ344469) and *A. dentiferum* (KP221815.1) given their close placement within Clade II (BS = 100%; Figure 6). In the initial evaluation, the grouping of these morphologically distinct taxa suggested potential biological phenomena such as incomplete lineage sorting (ILS), ancestral polymorphism, or reticulate evolution. However, this unusual topology is primarily considered to be an artifact of taxonomic misidentification within public repositories, where the sequence was incorrectly uploaded under the name *A. paniculatum*. The application of this name has historically been highly problematic due to an unclear taxonomic circumscription [58]. Following the revision of this mistakenly identified database sequence to A. cf. *paniculatum* in our ITS tree (Figure 6), it is suggested that this specific misidentification represents the primary source of the discrepancy observed in the topology regarding *A. yilandaghense* and *A. dentiferum*. This correction indicates that the positional conflict within the tree may not stem from actual evolutionary mechanisms like rapid speciation or lineage sorting, but rather from legacy nomenclatural errors in public sequence datasets.

A Kimura 2-parameter (K2P) genetic distance of 0.0632 (6.32%) exists between *A. aralii* and its closest morphological relative *A. turcicum* subsp. *turcicum* (Table 3). This distance is typical of interspecific variation in *Allium* (0.03–0.10) and greatly exceeds typical intraspecific variation (generally <0.01–0.02) [3,6]. Duchoslav et al. [41] identified that closely related species within the same clade of sect. *Codonoprasum* have genetic distances of 0.03 to 0.08, while major clades have distances > 0.10. The distance between *A. aralii* and *A. yilandaghense* (0.1125; Table 3) nears that of the interclade level, suggesting two species belong to evolutionarily discrete lineages, which is also supported by morphological differences (Table 1; Figure 1, Figure 2, Figure 3 and Figure 4).

Major conclusions regarding ITS data:

- *A. aralii* is genetically distinguishable from its morphologically closest relatives based on ITS sequence data, with pairwise distances ranging from 0.063 to 0.078, which fall within the typical interspecific range reported for *Allium* [3].
- High bootstrap support (90–100%) was recovered for the terminal clade containing *A. aralii* and its close relatives (*A. turcicum* subsp. *turcicum*, *A. euphraticum*, and *A. turcicum* subsp. *fusciflorum*), although deeper nodes within the eastern Mediterranean lineage showed only moderate support (BS = 59–64).
- The topology of our phylogenetic tree, which includes sequences from Clade I and II taxa (e.g., *A. daninianum*, *A. hermoneum*, *A. pseudostamineum*, *A. tauricola*, *A. rupestre*), is broadly consistent with the early diverging eastern Mediterranean lineages (Clades I–II) identified by Duchoslav et al. [41], although support values for deeper relationships remained moderate (BS = 59–64).

The matK dataset (15 taxa and 804 bp) supports less genetic differentiation between the taxonomically defined species studied; however, this information still provides useful genetic distance separation between species (Table 3). The K2P distance between *A. aralii* and *A. turcicum* subsp. *fusciflorum* = 0.0088 (0.88%) while distances between *A. euphraticum*, *A. yilandaghense*, and *A. turcicum* subsp. *turcicum* range from 0.0100 to 0.0113 (Table 4). According to Ipek et al. [57], matK is a slower evolving gene than ITS and is, thus, a more appropriate molecular marker for examining deeper phylogenetic relationships which will be reflected in the lower distances between closely related species.

The phylogeny provided in Figure 7, which was derived from matK, shows that *A. aralii* is closely related to *A. turcicum* subsp. *fusciflorum* ([94%] and [99%] bootstrap support, respectively). Therefore, the close relationship between *A. aralii* and *A. turcicum* subsp. *fusciflorum* as measured using matK, despite a greater degree of divergence from *A. turcicum* subsp. *fusciflorum* using ITS (0.0757 versus 0.0632), is one manifestation of an evolutionary process that has been documented by Duchoslav et al. [41].

Differences between ITS and matK topologies are widespread within *Allium* phylogenetic studies, especially within section *Codonoprasum*. Duchoslav et al. [41] have demonstrated that at least two processes occur frequently in this section: incomplete lineage sorting and chloroplast capture. However, hybridization and polyploidy may also contribute to topological discordance between markers, as suggested for other eastern Mediterranean taxa.

Southeastern Anatolia, especially around Bozova (Şanlıurfa), resides in the Irano-Turanian phytogeographical region, which has previously been recognized as a major center for *Allium* diversification and endemism [9,42]. The addition of *A. aralii* to the list of Middle-Eastern endemic *Allium* species is another example of how this area has become home to other relatively unstudied endemic species, including *A. euphraticum* [23], *A. yilandaghense* [25], and *A. turcicum* [25]. The abundance of narrow endemics supports the refugia-within-refugia hypothesis for the biodiversity hotspots of Anatolia.

Duchoslav et al. [41] note that while they assessed a more extensive number of taxa than any prior research, their sampling effort of Türkiye was minimal and they failed to analyze a significant portion of eastern or southeastern Anatolia—a poorly-documented floristic region. The research reported here fills this gap with molecular data for *A. aralii* and related species from Anatolia, and assists in enhancing our understanding of the importance of sect. *Codonoprasum*, in its center of endemism.

## 4. Materials and Methods

### 4.1. Morphology

In this study, about 20 specimens were collected in this study from type locality. For the identification of the collected specimens, the literature on *Allium* was reviewed and we consulted Flora of Türkiye and the East Aegean Islands. For the identification and comparison of the new species, the Flora of Türkiye and the Illustrated Flora of Türkiye, as well as the Floras of neighboring regions, were examined: Türkiye [9,10,59], Iran [60], Iraq [61], Lebanon [62], Syria, and Palestine [63], as well as historical references such as Post [64] and Boissier [65]. Morphological studies were carried out on fresh material of this *Allium* species using a stereo-binocular microscope, focusing on its description and diagnostic characters. Morphological data for the compared species (*A. turcicum* subsp. *turcicum*, *A. turcicum* subsp. *fusciflorum*, *A. euphraticum*, *A. yilandaghense*) were compiled from the literature [22,23,25]. The collected specimens are preserved in Harran University Faculty of Pharmacy Herbarium (HUEH) (Table 5).

Electron micrographs (scanning electron microscope, SEM) were obtained under a Zeiss EVO 50 SEM (Carl Zeiss Microscopy GmbH, Jena, Germany). at an accelerating voltage of 10 kV, three seeds were mounted onto aluminum stubs with double adhesive carbon tape and coated with 5 nm thick gold prior to observation. The descriptive terminology of seed surface sculpturing mainly follows Neshati and Fritsch [51], Choi and Cota-Sanchez [54], Bednorz et al. [52], Celep et al. [66], Choi et al. [52], Lin and Tan [53], Basanmunkh et al. [55], Yusuqov et al. [56], Khorasani et al. [67], and Shin et al. [68].

### 4.2. DNA Extraction, Amplification and Sequencing

Using the EurX GeneMATRIX Plant & Fungi DNA isolation kit (Gdańsk, Poland) and following the manufacturer’s directions, total genomic DNA was isolated from the silica gel-dried leaf tissue of *Allium aralii* and five comparison taxa (*A. euphraticum*, *A. turcicum* subsp. *turcicum*, *A. turcicum* subsp. *fusciflorum*, *A. yilandaghense*) for the purposes of molecular analysis. Both ITS (nuclear ribosomal internal transcribed spacer; using primers ITS1 and ITS4 [68]) and the matK gene (chloroplast; using primers 3F_KIM and 1R_KIM [69]) were selected as molecular markers to amplify [70]. PCR reactions were performed in 35 μL final volumes containing 6 μL of 2×Taq Plus PCR MasterMix (Solis Biodyne FIREPol^®^ DNA Polymerase, Tartu, Estonia), 3 μL of template DNA, 1.5 mM MgCl_2_, 0.3 μM of each primer, 0.2 mM dNTP mix and nuclease-free water. For the ITS region, the initial denaturation was for 5 min at 95 °C, followed by 30 denaturation cycles of 45 s at 95 °C, 45 s of annealing at 57 °C, and 60 s of extension at 72 °C, and a final extension of 5 min at 72 °C. For the matK region, the annealing was performed at 52 °C for 45 s instead. Amplified products were checked on 1% agarose gel electrophoresis and sent for bidirectional sequencing to BM Labosis (Ankara, Türkiye). A survey of ITS and matK–psbA accession numbers for newly sequenced accessions, as well as those downloaded from GenBank, is provided in Table 5.

### 4.3. Genetic Distance Analyses

Genetic divergence calculations among the taxa under investigation were calculated using the Kimura 2-parameter (K2P) model [49]) which accounts for the differing substitution rates between transition and transversion mutations. For both the ITS and matK datasets, distance matrices were created using MEGA 12.0 [71], and each ambiguous position was removed as a pairwise comparison was executed. Additionally, uncorrected p-distances were used for comparative purposes only. Pairwise interspecific genetic distances between *A. aralii* and its morphologically closest relatives (*A. euphraticum*, *A. turcicum* subsp. *turcicum*, *A. turcicum* subsp. *Fusciflorum*, and *A. yilandaghense*) were obtained from the resultant distance matrices.

### 4.4. Phylogenetic Tree Reconstruction

Maximum Likelihood (ML) phylogenetic analyses were carried out using MEGA 12.0 [71] based on the concatenated ITS + matK dataset comprising 5 taxa and 1304 aligned positions. The Tamura-Nei (TN93) model [72] with a gamma-distributed rate variation among sites (TN93+G) was chosen as the best-fitting model. The ML tree was inferred with 1000 bootstrap pseudo-replicates to evaluate nodal robustness. Bootstrap support (BS) values of 70% or higher were regarded as indicative of well-supported nodes [73]. Based on previous phylogenetic reconstructions [3,41], *Nothoscordum bivalve* (*GenBank accession OL537927.1*) was designated as the outgroup for the matK analysis, while *Allium ursinum* L. (sect. *Arctoprasum*) was selected as the outgroup for the ITS analysis.

## 5. Conclusions

The article describes of a new species, *Allium aralii* Balos, Köse & Sonay *sp.nov.*, found in southern Türkiye; morphological, seed micromorphological (using SEM), and molecular phylogenetic (using both ITS and matK) evidence all support the case that this species is a distinct member of *Allium* sect. *Codonoprasum*. Morphologically, *A. aralii* can be recognized by having blackish outer tunics; semi-cylindrical solid leaves exceeding the height of the inflorescence; an extraordinarily long persistent spathe; a goblet-shaped perigon (where the tepals are retained at maturity) with dentate inner tepals; bicolored stamens (whitish and purplish); a reticulate-foveate ovary; and verrucate ornamentation on the seed surfaces and undulate anticlinal walls. The genetic distances (using the ITS region) between *A. aralii* and its closest relatives range from 0.063, which falls within the expected range of genetic distances for *Allium* species when examined synoptically. *A. aralii* is known from only one site with <100 mature individuals, and therefore, according to IUCN criteria, it should be considered Critically Endangered (CR). The discovery of *A. aralii* emphasizes the southeastern portion of Türkiye as an underexplored area of *Allium* diversity and demonstrates the usefulness of integrating different types of data to clearly delineate species boundaries in taxonomically complex taxa.

## Figures and Tables

**Table 1:** Distinguishing characters between *A. aralii*, *A. euphraticum* [23], *A. turcicum* subsp. *turcicum* [22], *A. turcicum* subsp. *fusciflorum* [22], and *A. yilandaghense* [25].

Character	*A. aralii*	*A. euphraticum*	*A. turcicum* subsp. *turcicum*	*A. turcicum* subsp. *fusciflorum*	*A. yilandaghense*
Bulb	1.0–1.5 cm, blackish outer tunics	1–2 cm, brownish outer tunics	1–2 cm	1–2 cm, brownish-black outer tunics	2.5–3 cm, light brown outer tunics
Bulblets	Absent	Absent	Absent	Absent	Present (1–3)
Scape	16–30 cm, slender	14–44 cm	10–40 cm	10–40 cm	16–30 cm, upper part bent
Leaves	Semi-cylindrical, solid, exceeding inflorescence	Filiform, hollow, exceeding inflorescence	Semi-cylindrical	Semi-cylindrical	Flat to semi-cylindrical, not exceeding inflorescence
Spathe	Very long, strongly unequal	Long, unequal	Long	Long	Equal to or longer than inflorescence
Inflorescence	Lax, hemispherical	Lax	Lax	Lax	Lax, wider
Perigon shape	Goblet-shaped	Campanulate	Campanulate	Globose	Campanulate
Tepal color	Brownish to greenish	Cream to brownish	Pale	Brownish	Yellowish-green
Tepal apex	Inner tepals dentate	Mucronate	Irregularly dentate	Irregularly dentate	Obtuse
Stamens	Exserted, short filaments (3.5–4.0 mm)	Exserted, 5.5–6.0 mm	Exserted	Very long (≈2× tepals)	Exserted
Filament color	White with purple middle	Upper part purplish	Upper part purplish	Upper part purplish	Upper part purplish
Ovary	Reticulate-foveate	Reticulate-papillose	Reticulate	Papillose	Reticulate-papillose
Style	3.5–5.5 mm	1.5–5.5 mm	3.5–4.0 mm	2.0–5.8 mm	5.0–5.5 mm
Capsule	Globose	Globose	Globose	Globose	Globose
Seed shape	Hemispheroidal	Oblong	Sub-oblong	Oblong	Subovate, flattened

**Table 2:** Comparison of seed surfaces of *Allium aralii* and related species.

Character	*Allium aralii*	*Allium euphraticum*	*Allium turcicum*	*Allium fusciflorum*	*Allium yilandaghense*
Seed shape	Oval-elliptic	Broadly oval	Elongated-elliptic	Ovate-oblong	Elliptic-oblong
Seed size (L × W)	2.8–3.2 × 1.4–1.6 mm	3.1–3.5 × 1.9–2.2 mm	2.5–3.0 × 1.2–1.4 mm	2.9–3.3 × 1.5–1.7 mm	3.0–3.4 × 1.6–1.8 mm
Testa cell shape	Polygonal-elongated	Broadly polygonal	Narrowly elongated	Oblong-polygonal	Hexagonal-oblong
Cell arrangement	Longitudinal	Irregular-longitudinal	Strictly longitudinal	Longitudinal	Longitudinal
Cell dimensions (L × W) (µm)	45.5–62.0 × 22.0–34.5	42.0–58.5 × 24.5–36.0	38.5–54.0 × 20.0–32.5	44.0–60.0 × 23.0–35.0	40.0–56.0 × 21.5–33.5
Cell boundary (Anticlinal)	Undulate	Sinuate-undulate	Straight to curved	Undulate-sinuate	Sinuate
Anticlinal wall type	Thickened	Strongly thickened	Thin	Medium thickened	Thickened
Anticlinal wall width (µm)	3.2–4.8	3.5–5.2	2.5–4.0	3.0–4.5	3.4–5.0
Periclinal wall	Convex	Strongly convex	Flat to subconvex	Convex	Subconvex
Ornamentation	Verrucate	Granular-verrucate	Smooth-striate	Verrucate	Rugose-verrucate
Verrucae	Present (Small)	Present (Large)	Absent/Indistinct	Present	Present
Cell boundaries	Distinct	Very distinct	Moderately distinct	Distinct	Distinct
Intercellular distance (µm)	1.5–2.5	2.8–4.5	1.2–2.4	1.8–3.2	2.0–3.5

**Table 3:** Kimura 2-parameter (K2P) genetic distances (below diagonal) based on ITS sequences for *A. aralii* and related taxa within sect. *Codonoprasum*.

	Taxon	1	2	3	4	5	6	7	8
1	*A. aralii* (PZ344467)	-							
2	*A. turcicum* subsp. *turcicum* (PZ344470)	0.063	-						
3	*A. euphraticum* (PZ344471)	0.072	0.027	-					
4	*A. stamatiadae* (OR116805)	0.074	0.065	0.078	-				
5	*A. turcicum* subsp. *fusciflorum* (PZ344468)	0.076	0.035	0.037	0.088	-			
6	*A. gilanense* (MT303159)	0.076	0.078	0.087	0.031	0.102	-		
7	*A. parciflorum* (KP221829)	0.078	0.072	0.082	0.051	0.093	0.041	-	
8	*A. paniculatum* (MK776928)	0.078	0.069	0.072	0.055	0.088	0.061	0.061	-

**Table 4:** Kimura 2-parameter (K2P) genetic distances (below diagonal) based on *matK* sequences for *A. aralii* and related taxa within sect. *Codonoprasum*.

Taxon	1	2	3	4	5	6	7
1. *A. aralii*	-						
2. *A. turcicum* subsp. *fusciflorum*	0.0088	-					
3. *A. yilandaghense*	0.0100	0.0012	-				
4. *A. turcicum* subsp. *turcicum*	0.0113	0.0025	0.0037	-			
5. *A. euphraticum*	0.0100	0.0012	0.0025	0.0037	-		
6. *A. nigrum* (NC_057585.1)	0.0100	0.0138	0.0151	0.0164	0.0151	-	
7. *A. oleraceum* subsp. *oleraceum* (OR884984.1)	0.0126	0.0164	0.0177	0.0189	0.0177	0.0025	-

**Table 5:** Collection localities, voucher specimens, and GenBank accession numbers of Allium taxa analyzed for ITS and matK regions.

Species	Accession Number	Sect.	Locality
***A. aralii***	PZ344467	*Codonoprasum*	(Şanlıurfa: Türkiye) Şanlıurfa: Bozova, Mt. Kaplandağı, oak and rocky area, 37°20′0.25″ N, 38°34′11.06″ E, 840 m, 25 May 2024, M. Balos 5616 (holotype: HUEH; isotypes: ESSE).
***A. turcicum*** **subsp. *fusciflorum***	PZ344468	*Codonoprasum*	(Mardin: Türkiye) Mardin Province, Derik region, GAP valley, rocky slopes, 37°21′46.90″ K, 40°17′54.88″ D, 940 m, 7 May 2025, M. Balos 5668 (HUEH).
***A. yilandaghense***	PZ344469	*Codonoprasum*	(Batman: Türkiye) Batman, Sason, Sason mountains, between the villages of Çalışırlar and Taşyuva, 38°22′11.55″ K, 41°31′31.39″ D, 2215 m., hills, steppe, 14 June 2025, M. Balos 5715.
***A. turcicum*** **subsp. *turcicum***	PZ344470	*Codonoprasum*	(Batman: Türkiye) Batman, Sason, Sason mountains, between the villages of Çalışırlar and Taşyuva, 38°22′21.10″ K, 41°29′23.61″ D, 1494 m., roadside, steppe area, 14 June 2025, M. Balos 5714.
***A. euphraticum***	PZ344471	*Codonoprasum*	(Elazığ: Türkiye) Elazığ Province, Harput district, Serince village, roadside, steppe area, 1270 m a.s.l., 22 June 2023, M. Balos 5502 & V. Sonay, Y. Yeşildal (holotype HARRAN, isotype HARRAN).
***A. parciflorum***	KP221829.1	*Codonoprasum*	
***A. gilanense***	MT303159.1	*Codonoprasum*	
***A. paniculatum***	MK776928.1	*Codonoprasum*	
***A. stamatiadae***	OR116805.1	*Codonoprasum*	
***A. marginatum***	OR116810.1	*Codonoprasum*	
***A. fuscum***	PP834289.1	*Codonoprasum*	
***A. oleraceum***	FJ664286.1	*Codonoprasum*	
***A. goumenissanum***	OR116786.1	*Codonoprasum*	
***A. dentiferum***	KP221815.1	*Codonoprasum*	
***A. castellanense***	KP221825.1	*Codonoprasum*	
***A. flavum***	OM891951.1	*Codonoprasum*	
***A. melanantherum***	AJ412739.1	*Codonoprasum*	
***A. praescissum***	OM891954.1	*Codonoprasum*	
***A. lenkoranicum***	MT303162.1	*Codonoprasum*	
***A. flavum var. minus***	AJ411926.1	*Codonoprasum*	
***A. tardans***	KP221823.1	*Codonoprasum*	
***A. rupestre***	PX999857.1	*Codonoprasum*	
***A. tauricola***	PX999870.1	*Codonoprasum*	
***A. daninianum***	PX999767.1	*Codonoprasum*	
***A. hermoneum***	PX999807.1	*Codonoprasum*	
***A. pseudostamineum***	PX999838.1	*Codonoprasum*	
***A. ursinum***	FR682005.1	*Arctoprasum*	

## Data Availability

The original contributions presented in this study are included in the article. Further inquiries can be directed to the corresponding author.
